# Epigenetic regulation of gene expression and cellular differentiation

**DOI:** 10.1186/1744-8069-10-S1-O20

**Published:** 2014-12-15

**Authors:** Artem Barski, Suresh Cuddapah, Andrey V Kartashov, Hiromi Imamichi, Wenjing Yang, Weiqun Peng, H Clifford Lane, Keji Zhao

**Affiliations:** 1 Cincinnati Children's Hospital Medical Center, Cincinnati, OH 45229, USA; 2 New York University, New York, NY 10012, USA; 3 National Institute of Allergy and Infectious Diseases, NIH, Bethesda, MD, USA; 4 George Washington University, Washington, DC 20052, USA; 5 National Heart, Lung and Blood Institute, NIH, Bethesda, MD 20892, USA

## 

Epigenome is defined by a collection of various chromatin modifications, which maintains a chromatin environment that is either permissive or inhibitory for gene expression. While association of histone modifications with expressed or silent genes has been established, it remains unclear how changes in chromatin modifications relate to changes in gene expression. We used ChIP-Seq to analyze the genome-wide changes in chromatin modifications during short-term activation of naïve and memory CD4+ T cells by T cell receptor (TCR) signaling. In resting and activated T cells, expressed genes were strongly associated with “active” modifications (e.g. H3K4me1/2/3, H2A.Z) and RNA Polymerase II (Pol II), while silent genes were typically associated with repressive marks. However, we found that about 20-30% of silent genes were poised; they possessed positive modifications and sometimes even Pol II at their promoters. Interestingly, majority of genes induced during T cell activation were poised in resting T cells even before the TCR signaling was initiated. Our data suggest that T cell memory in which immunological memory state is determined epigenetically-by transcriptional memory and poising.

